# Ligand Mobility Modulates Immunological Synapse Formation and T Cell Activation

**DOI:** 10.1371/journal.pone.0032398

**Published:** 2012-02-22

**Authors:** Chih-Jung Hsu, Wan-Ting Hsieh, Abraham Waldman, Fiona Clarke, Eric S. Huseby, Janis K. Burkhardt, Tobias Baumgart

**Affiliations:** 1 Department of Chemistry, The Children's Hospital of Philadelphia and Perelman School of Medicine, University of Pennsylvania, Philadelphia, Pennsylvania, United States of America; 2 Department of Pathology and Laboratory Medicine, The Children's Hospital of Philadelphia and Perelman School of Medicine, University of Pennsylvania, Philadelphia, Pennsylvania, United States of America; 3 Department of Pathology, University of Massachusetts Medical School, Worcester, Massachusetts, United States of America; University of Cambridge, United Kingdom

## Abstract

T cell receptor (TCR) engagement induces clustering and recruitment to the plasma membrane of many signaling molecules, including the protein tyrosine kinase zeta-chain associated protein of 70 kDa (ZAP70) and the adaptor SH2 domain-containing leukocyte protein of 76 kDa (SLP76). This molecular rearrangement results in formation of the immunological synapse (IS), a dynamic protein array that modulates T cell activation. The current study investigates the effects of apparent long-range ligand mobility on T cell signaling activity and IS formation. We formed stimulatory lipid bilayers on glass surfaces from binary lipid mixtures with varied composition, and characterized these surfaces with respect to diffusion coefficient and fluid connectivity. Stimulatory ligands coupled to these surfaces with similar density and orientation showed differences in their ability to activate T cells. On less mobile membranes, central supramolecular activation cluster (cSMAC) formation was delayed and the overall accumulation of CD3ζ at the IS was reduced. Analysis of signaling microcluster (MC) dynamics showed that ZAP70 MCs exhibited faster track velocity and longer trajectories as a function of increased ligand mobility, whereas movement of SLP76 MCs was relatively insensitive to this parameter. Actin retrograde flow was observed on all surfaces, but cell spreading and subsequent cytoskeletal contraction were more pronounced on mobile membranes. Finally, increased tyrosine phosphorylation and persistent elevation of intracellular Ca^2+^ were observed in cells stimulated on fluid membranes. These results point to ligand mobility as an important parameter in modulating T cell responses.

## Introduction

Cell membranes have unique physical properties including lateral heterogeneity, fluid nature, and diverse surface topology. Signal transduction across the plasma membrane is commonly accompanied by the coordinated reorganization of membrane components. This is illustrated by the interaction between T cells and antigen presenting cells (APCs). Within minutes of cell-cell contact, signaling components at the cell-cell interface assemble into signaling MCs, which then undergo a large-scale spatial rearrangement to form an ordered array termed the immunological synapse (IS) [1], [2], [3]. MCs containing TCR with its associated CD3 signaling chains move centripetally and coalesce into a structure known as the central supramolecular activation cluster (cSMAC), while adhesion molecules segregate to form an outer ring, termed the peripheral supramolecular activation cluster (pSMAC) [2], [4]. After formation of IS, TCR-proximal MCs are continuously assembled near the cell periphery and translocate to the cSMAC region [5]. These newly-generated TCR MCs are essential for the maintenance of IS structure [3] and for sustained signaling [5]. It is generally believed that MCs serve as primary signaling sites where early tyrosine phosphorylation events take place, leading to downstream signaling events such as calcium flux and transcriptional activation [5], [6], [7].

Signaling MCs are composed of numerous proteins, including TCRs, kinases, and adaptor molecules [5], [6], [7]. TCR engagement leads to phosphorylation of immunoreceptor tyrosine-based activation motifs (ITAMs) within the CD3 complex by the Src family kinase Lck. These phosphorylated ITAMs serve as docking sites for the tyrosine kinase ZAP70, which in turn phosphorylates several adaptor proteins including linker for activation of T cells (LAT) and SLP76 [5], [8], [9]. These adaptors contain multiple protein binding domains and function to mediate the assembly and localization of signaling complexes. During their centralization, MC components have been observed to exhibit distinct behaviors and to segregate from each other [5]. However, the transport mechanisms regulating MC movement are poorly understood, and the basis for molecular segregation is still debated.

MC dynamics and IS topology differ depending on the type of APC with which a T cell interacts, and some, but not all, APCs exhibit polarization of actin and other cytoskeletal elements toward the IS. In particular, dendritic cells (DCs) recruit F-actin and the actin binding protein fascin to the site of T cell engagement [10], and this process is required for IS formation and T cell activation [11]. Similar events have recently been described in DCs engaging natural killer cells [12]. Although it has not been directly demonstrated, it is likely that the rearrangement of the DC cortical cytoskeleton affects ligand mobility at the synaptic interface. In other systems, ligand mobility is an important parameter for cellular activation. In RBL cells, for example, formation of the mast cell synapse depends on ligand lateral mobility [13], [14]. Despite its possible physiological significance, the possibility that T cell activation is also modulated by ligand mobility at the synaptic interface has not been directly addressed.

Much of what is known about molecular dynamics at the IS has been derived from studies using solid-supported platforms to mimic the stimulatory surfaces of APCs [15]. Ligands are typically either immobilized completely on glass coverslips [8], [16], or attached to extremely mobile single-component bilayer surfaces [4], [17], [18]. These two systems represent extremes of mobility, both of which are outside the physiological range. Side-by-side comparisons are rarely made, and differences in surface chemistry and methods of ligand immobilization complicate interpretation of such studies. Most importantly, neither experimental system permits the systematic exploration of the influence of ligand mobility on T cell signaling.

In this contribution, we have used planar lipid bilayers composed of a binary mixture of 1,2-dimyristoyl-*sn*-glycero-3-phosphocholine (DMPC) and 1,2-dipalmitoyl-*sn*-glycero-3-phosphocholine (DPPC). By varying the proportion of these two lipids, we could vary membrane fluidity and heterogeneity, characterizing the long-range apparent ligand mobility by the lateral diffusion coefficient *D* and fluorescence recovery after photobleaching (FRAP) recovery fraction *R*. We demonstrate that the dynamics of cSMAC formation, MC movement, and actin contraction were all modulated by apparent ligand mobility. Under the stimulatory conditions tested here, we found that T cell activation, as measured by elevated levels of tyrosine phosphorylation and sustained intracellular calcium influx was preferentially induced by more fluid membranes. Our data demonstrate that ligand mobility is an important physiological parameter that modulates MC transport and T cell activation.

## Results

### Generation and characterization of supported lipid bilayers with variable ligand mobility

**Figure 1:**
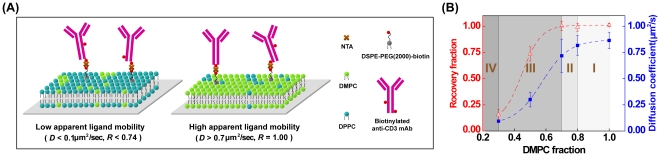
A variety of membrane fluidities and heterogeneities on the binary mixture of lipid bilayers. (A) Schematic illustration of self-assembled supported lipid bilayers containing a mixture of DMPC, DPPC, and 2% DSPE-PEG2000-biotin. Biotinylated anti-CD3 monoclonal antibody was anchored to bilayer surfaces via neutravidin (NTA). (B) The diffusion coefficient *D* (blue solid squares) and mobile fraction *R* (red open triangles) of NTA were determined by curve-fitting to the traces of fluorescence recovery after photobleaching at 37°C. The graph was further divided into four regions: I, fluid phase region; II, fluid-connected region; III, fluid-confined region; and IV, low mobility region. Dashed lines are sigmoidal curve fits to guide the eye. Error bars represent the standard error of the mean. N>6 FRAP measurements.

Previous analysis of molecular dynamics at the immunological synapse has utilized either coverslips coated with immobile ligands or planar lipid bilayers under high mobility conditions. However ligands in biological membranes exhibit intermediate mobilities, which vary depending on cell physiology [19]. We therefore sought to generate bilayers with a wide range of membrane fluidities (which determine ligand mobilities) at 37°C. To achieve this, we used a mixture of DMPC (gel/fluid transition temperature Tm = 23°C) and DPPC (Tm = 41°C). By simply varying the ratio of these two lipids, the long range ligand diffusion coefficient can be modulated over nearly a 10-fold range. Equally importantly, for this lipid combination a well-characterized phase diagram is known that displays a gel-fluid coexistence region at physiological temperature [20], [21]. Biotinylated anti-CD3 antibody (clone OKT3) was linked to membrane-embedded DSPE-PEG(2000)-biotin via neutravidin (NTA). We chose NTA over streptavidin as a coupling agent because the former shows significantly reduced aggregation on the bilayer at high protein concentration [22], [23], [24]. Fig. 1A shows a schematic of our experimental system.

To characterize the properties of these surfaces, NTA long-range diffusion coefficients (*D*) and recovery fraction (*R*) were measured by FRAP at 37°C (Fig. 1B and Fig. S1) [25]. In a mixed lipid system with gel/fluid phase coexistence, long-range diffusion is quantitatively described by percolation theory, where lateral translocation is highly influenced by the presence of gel domains and the connectivity between fluid domains [26], [27]. As gel phase domains become more abundant, long range diffusion is progressively hindered. Eventually, once the mixture reaches the percolation threshold, the fluid phase is substantially surrounded by gel domains, and connectivity of the fluid phase is interrupted [26], [27]. In our system, FRAP measurements demonstrated the existence of a single fluid phase in bilayers containing 80% DMPC and 20% DPPC; above this level of DMPC, both *D* and *R* remained relatively constant (Fig. 1B). At this composition, the diffusion coefficient of NTA was comparable to other proteins anchored on fluid membranes [28]. At 70% DMPC, *D* decreased slightly due to the presence of gel phase domains (see below), but *R* was nearly complete (i.e. close to one), indicating that fluid phase regions remained connected (Fig. 1B). Under these conditions, the constrained gel domains are surrounded by the fully recovered fluid area, suggesting that NTA molecules anchored via PEG(2000)-biotin preferentially partition into the fluid region of the gel-fluid coexistence phase. Between 70% and 30% DMPC, dramatically descending *R* and *D* were found, presumably resulting from the increase of the gel phase fraction. We note that compared to the published phase diagrams [20], [21], the phase transition temperature in our system was increased by NTA binding (see below). A similar effect has been shown for cholera toxin subunit B (CTB) binding on monosialoganglioside GM1 embedded in DMPC membranes [29], although the underlying mechanisms might be different.

Based on FRAP measurements, the apparent ligand mobility in our bilayer system can be divided into four regions according to DMPC content, *D*, and *R*. These regions are diagrammed in Fig. 1B. Within the fluid phase region (Region I, DMPC content greater than 80%), ligands can freely diffuse laterally, exhibiting *D* varying from 0.82 to 0.86 µm^2^/sec, and *R* = 1. In the fluid-connected region (Region II, 70–80% DMPC), lateral diffusion is hindered by the co-existence of gel phase nanodomains but the fluid phase connectivity is still high, *D* ranged from 0.72 to 0.82 µm^2^/sec, and *R* = 1. In the fluid-confined region (Region III, DMPC content between 30–70%), the fluid phase is isolated by the increasing gel phase domains, *D* from 0.09 to 0.72 µm^2^/sec, and 0.16≤R≤0.74. Finally, in the low mobility region (Region IV, DMPC content <30%), long-range lateral translocation is severely obstructed, *D*<0.09 µm^2^/sec, and R<0.16.

For certain lipid mixing ratios, phase separation of the lipid mixture leads to the formation of compositional domains within a bilayer. Atomic force microscopy (AFM), which can scan surfaces for topological differences on an atomic scale, was used to characterize the homogeneity of our surfaces, and to ask what influence addition of NTA has on lipid domain organization [30], [31]. At 37°C, the membrane surface containing 70% DMPC was uniform, but became heterogeneous after the addition of NTA (Fig. S2). The segregated areas were below 300 nm in diameter, i.e., not resolvable with optical microscopy (Fig. S1A). To test whether the amount of protein coupled to the membrane could be affected by the surface heterogeneity, coupled anti-CD3 antibody was labeled with fluorescent anti-mouse antibody to monitor ligand density. We observed comparable fluorescence intensities of bilayers made at all four DMPC∶DPPC ratios, indicating that the number of anchored ligands on different bilayer surfaces was not significantly influenced by the presence of surface heterogeneity (Fig. S3).

### Apparent ligand mobility modulates cSMAC formation

**Figure 2:**
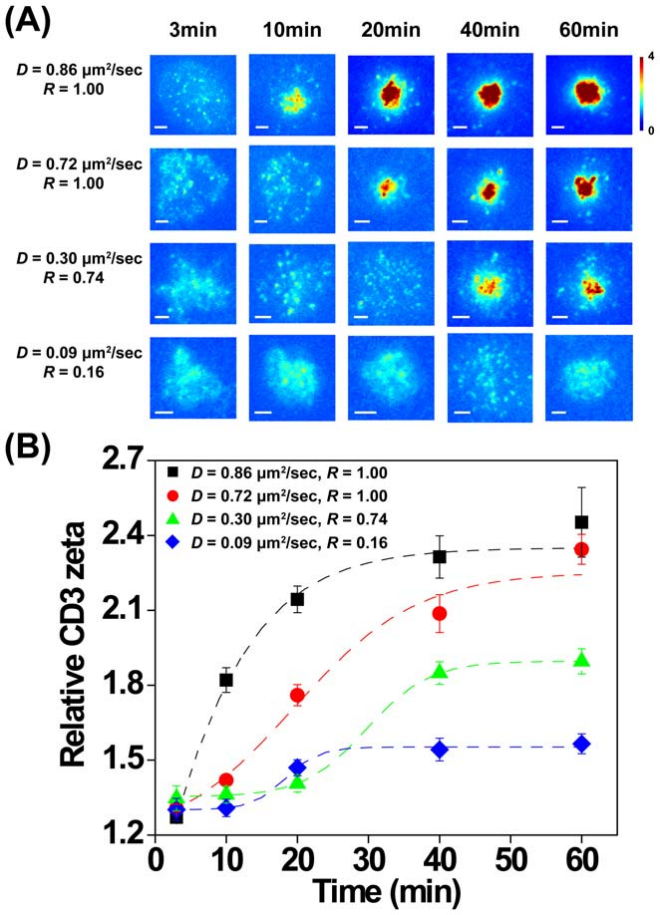
Ligand mobility modulates cSMAC formation and CD3ζ accumulation. Jurkat T cells were allowed to interact with stimulatory surfaces for the indicated times, after which cells were fixed and labeled for CD3ζ. (A) Representative TIRF images showing relative accumulation of CD3ζ, are depicted using a heat map. Scale bars = 3 µm. (B) Relative CD3ζ accumulation in cells stimulated on the indicated membranes, plotted as a function of time. Dashed lines are sigmoidal curve fits to guide the eye. Error bars represent the standard error of the mean. N>30 cells per condition.

T cells stimulated with surfaces coated with high-mobility ligands exhibit centripetal movement of TCR MCs, to form the cSMAC region of the IS. This movement requires ligand mobility; central accumulation of TCR is not observed in cells stimulated by surface-immobilized ligands [9] or movement-limited ligands [32]. To better understand the effects of varying ligand mobility on the dynamics of cSMAC formation, we investigated the distribution of CD3ζ in T cells stimulated on our panel of mixed-lipid surfaces. Jurkat T cells were allowed to interact with OKT3-coated surfaces for different times, fixed, and stained with fluorescent anti-CD3 antibody. The relative accumulation of CD3ζ at the IS was then determined based on the ratio of immunofluorescence intensity over the entire cell-surface contact to the intensity of a background field. As shown in Fig. 2, the distribution and level of CD3ζ were similar on all stimulatory membranes at the 3 minute time point. At later times, however, clear differences were observed among the different stimulatory surfaces. T cells engaging the fluid membrane (*D* = 0.86 µm^2^/sec, *R* = 1.00), exhibited clear central accumulation of CD3ζ, by 10 minutes of contact, consistent with previous observations on high mobility planar lipid bilayers [5], [18]. CD3ζ continued to accumulate in the IS region, reaching a plateau by 40 minutes at ∼2.5-fold over background. This structure existed stably for at least one hour. On the other extreme, T cells stimulated on the low mobility membrane (*D* = 0.09 µm^2^/sec, *R* = 0.16) did not exhibit cSMAC formation, and CD3ζ remained randomly distributed across the contact interface, in keeping with studies using ligands immobilized on glass coverslips [8]. On the fluid-connected surfaces (*D* = 0.72 µm^2^/sec, *R* = 1.00), cSMAC formation was delayed to 20 min (Fig. 2A), and on fluid-confined membranes (*D* = 0.30 µm^2^/sec, *R* = 0.74) cSMAC formation was slowed to 40 min, in parallel with lower relative CD3ζ accumulation (Fig. 2B). This suggests that the lateral transport of TCR/CD3 was impeded by the presence of gel domains. Taken together, these results demonstrate that the rate and extent of CD3ζ centralization is modulated by variable ligand mobility and fluid connectivity.

### Apparent ligand mobility differentially modulates MC translocation

Upon TCR ligation, key signaling intermediates including the tyrosine kinase ZAP70 and molecular adaptor SLP76 are recruited into signaling complexes at the plasma membrane. These complexes are visible as signaling MCs that assemble near the periphery of the spreading cell and move centripetally toward the cSMAC. To investigate the influence of *D* on T cell signaling MCs, Jurkat T cells expressing ZAP70-GFP or SLP76-GFP were stimulated on supported membranes with different *D* at 37°C. The spatial and temporal positions of signaling MCs were used to compute MC track velocity (the total distance traveled divided by the time required to travel that distance) and net MC displacement (the shortest distance between the position where a MC appeared and the position where it disappeared).

**Figure 3:**
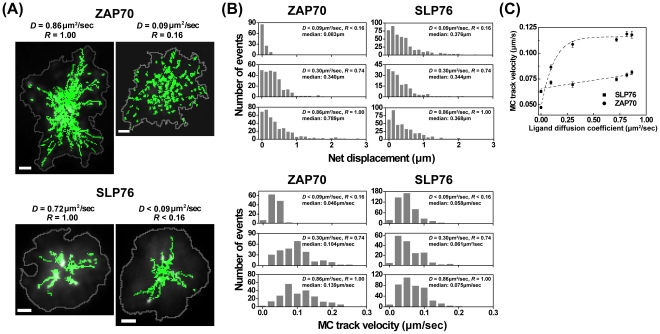
Ligand mobility modulates translocation of ZAP70 and SLP76 MCs. Jurkat T cells expressing GFP-ZAP70 or GFP-SLP76 were imaged by TIRF microscopy while interacting with OKT3-coated bilayer surfaces with different ligand mobility (A) MC trajectories (green) were tracked for ZAP70 (left) and SLP76 (right). Cell boundaries are depicted as gray lines on representative cell images. Scale bars = 2 µm. (B) Histogram analysis of net displacement (top) and MC track velocity (bottom) for ZAP70 (left) or SLP76 (right) on stimulatory bilayer surfaces with indicated *D* and *R*. (C) Average track velocity for MCs containing ZAP70 (circles) or SLP76 (squares), analyzed as a function of ligand diffusion coefficient. Dashed lines are sigmoidal curve fits. Error bars represent the standard error of the mean. N>6 cells per condition.

As shown in Figure 3, the movement of ZAP70 MCs was dramatically affected by ligand mobility. On the fluid membrane, centripetally moving ZAP70 MCs displayed long trajectories, most of which extended from the site of MC formation in the periphery to the cSMAC region (Fig. 3A, top, and Supplemental Videos S1 and S2). In contrast, T cells interacting with low mobility ligands showed highly confined movement of ZAP70 MCs. Quantitative analysis of MC movement (Fig. 3B, left) shows that on the low mobility ligand surfaces (*D*<0.09 µm^2^/sec, *R*<0.16), most ZAP70 MCs are stationary; these tracking data are comparable to those of cells stimulated by glass-coated ligands (data not shown). Comparing the fluid membranes (*D* = 0.86 µm^2^/sec, *R* = 1.00) with the confined fluid surfaces (*D* = 0.30 µm^2^/sec, *R* = 0.74), the fraction of ZAP70 MCs with long net displacement and fast track velocity is highly increased with increased ligand mobility.

In contrast with ZAP70 MCs, SLP76 MCs showed centripetal movement even on low mobility bilayers (Fig. 3A, right, and Supplemental Video S3). With increased ligand mobility, SLP76 MCs displayed a marginal enhancement in track velocity and net displacement (Fig. 3B, right, and Supplemental Video S4). The relationship between ligand diffusion coefficient *D* and MC track velocity for ZAP70 and SLP76 MCs is shown in Fig. 3C. This analysis reveals that although ZAP70 MCs were less dynamic than SLP76 MCs on the low mobility membrane, ZAP70 track velocity quickly increased as a function of *D*, surpassed SLP76 track velocity, and reached a plateau of 0.109–0.119 µm/sec. This shows that ZAP70 MCs are more responsive to ligand mobility than SLP76 MCs.

**Figure 4:**
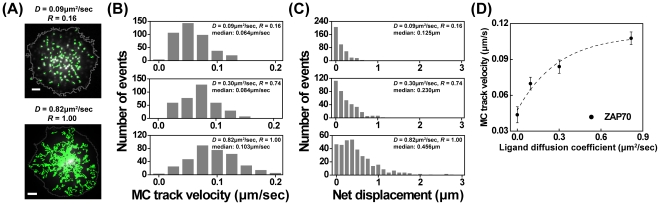
Ligand mobility modulates ZAP70 MC translocation in primary mouse T cells. CD4+ mouse T cell blasts were transduced with GFP-ZAP70, and imaged by TIRF microscopy while interacting with 2C11-coated bilayer surfaces with different ligand mobility. (A) Representative images showing tracking of MC trajectories (green). Cell boundaries are depicted as gray lines. Scale bars = 2 µm. (B,C) Histogram analysis of MC track velocity (B) and net displacement (C) on stimulatory bilayer surfaces with indicated *D* and *R*. (D) Average MC track velocity plotted as a function of ligand diffusion coefficient. Dashed line is sigmoidal curve fit to guide the eye. N>5 cells per condition.

The studies above were conducted using Jurkat T cells, a well-characterized line that facilitates imaging studies. To verify that MC dynamics are modulated by ligand mobility in primary T cells, mouse CD4+ T cell blasts were transiently transfected with ZAP70-GFP and MC translocation was analyzed. Because primary T cells require ligation of integrins as well as TCR to undergo spreading on planar surfaces, bilayers were loaded with both biotinylated anti-mouse CD3 antibody (clone 2C11) and biotinylated mouse ICAM-1. As expected, bilayers loaded with ICAM1 alone were sufficient to promote adhesion, but TCR engagement was needed to initiate T cell activation as measured by tyrosine phosphorylation and ZAP70 MC formation (Fig. S4). As observed in Jurkat cells, ZAP70 MCs in primary T cells were nucleated at the cell periphery and translocated centripetally on fluid bilayer surfaces, but tended to remain stationary on low mobility surfaces (Fig. 4A, and Supplemental Videos S5 and S6). As shown in Fig. 4B and C, ZAP70 MCs exhibited faster track velocity and longer net displacement with increasing ligand mobility. Analysis of MC track velocity as a function of *D* shows that in primary mouse T cells, as in Jurkat T cells, ZAP70 MC movement is highly related to *D* (Fig. 4D).

### Actin distribution is altered by apparent ligand mobility

**Figure 5:**
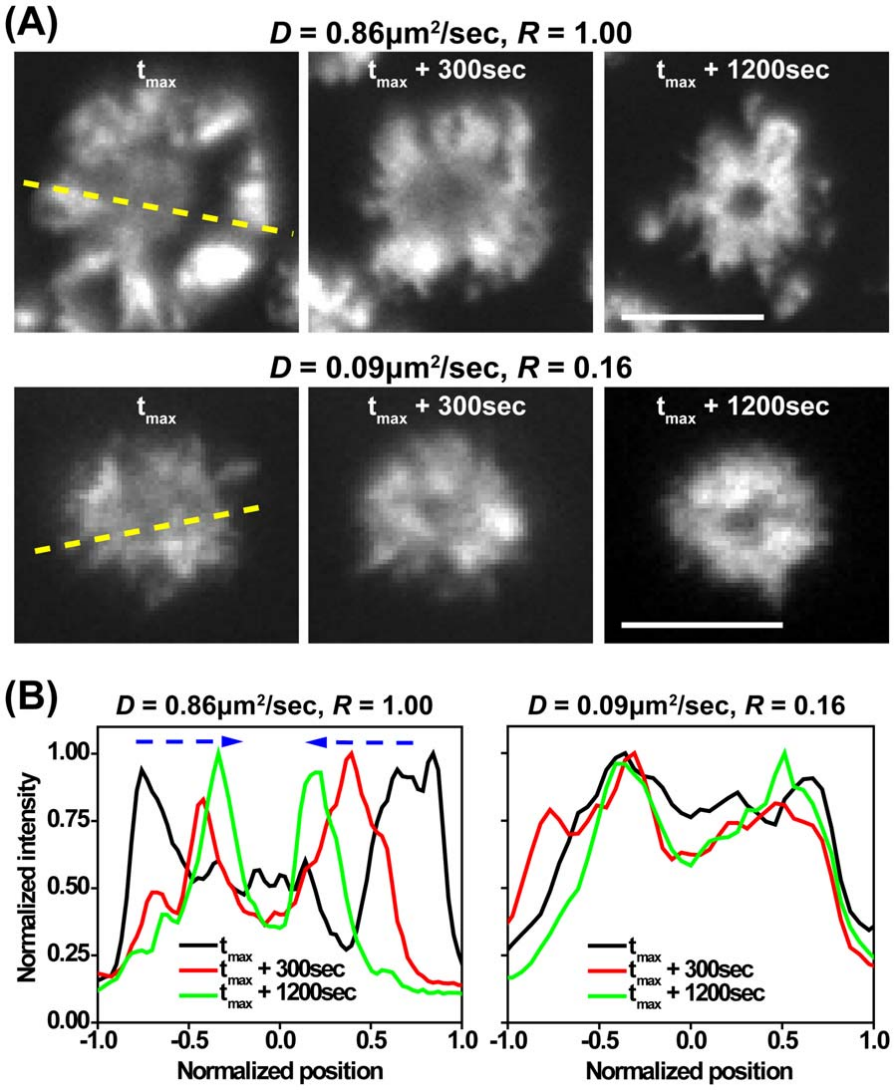
Actin contraction is altered by ligand mobility. (A) Representative time-lapse TIRF images of GFP-actin expressing Jurkat cells stimulated on surfaces with the indicated *D* and *R*. The time when cells exhibit the maximum actin spreading was defined as t_max_. Scale bars = 10 µm. (B) Corresponding normalized actin fluorescence intensities at t_max_ (black), t_max_+300 sec (red), and t_max_+1200 sec along the dashed lines in (A).

The central translocation of MCs is driven at least in part by actin retrograde flow [8], [33]. Although actin dynamics have been extensively studied in cells either activated on bilayers or on ligand-coated glass surfaces, there have been no side-by-side comparisons to elucidate the influence of ligand mobility on the actin cytoskeleton. To address this question, Jurkat cells stably expressing GFP-actin were activated on stimulatory bilayer surfaces with varying ligand mobility and the distribution of GFP-actin was tracked. On both high mobility and low mobility membranes coated with stimulatory ligands, T cells quickly spread and extended lamellipodial protrusions. After spreading was maximal, the cells exhibited high levels of actin within the lamellipodium, and an actin-poor central region corresponding roughly to the cSMAC region (Fig. 5A). Interestingly, T cells spreading on fluid membranes (*D* = 0.86 µm^2^/sec, *R* = 1.00), exhibited contraction of the actin-rich region within 5 minutes after the time of maximal spreading (Fig. 5B left). In contrast, cells stimulated on the low mobility membrane (*D* = 0.09 µm^2^/sec, *R* = 0.16), became somewhat less spread and showed less subsequent contraction, such that the distribution of the actin network remained relatively constant (Fig. 5B right). Actin retrograde flow occurred on all stimulatory membranes, and was persistent for at least one hour (data not shown). Thus, we find that variable ligand mobility has comparatively little effect on actin retrograde flow, but does impact the contractility of the actin network.

### Ligand mobility impacts the efficiency of T cell activation

**Figure 6:**
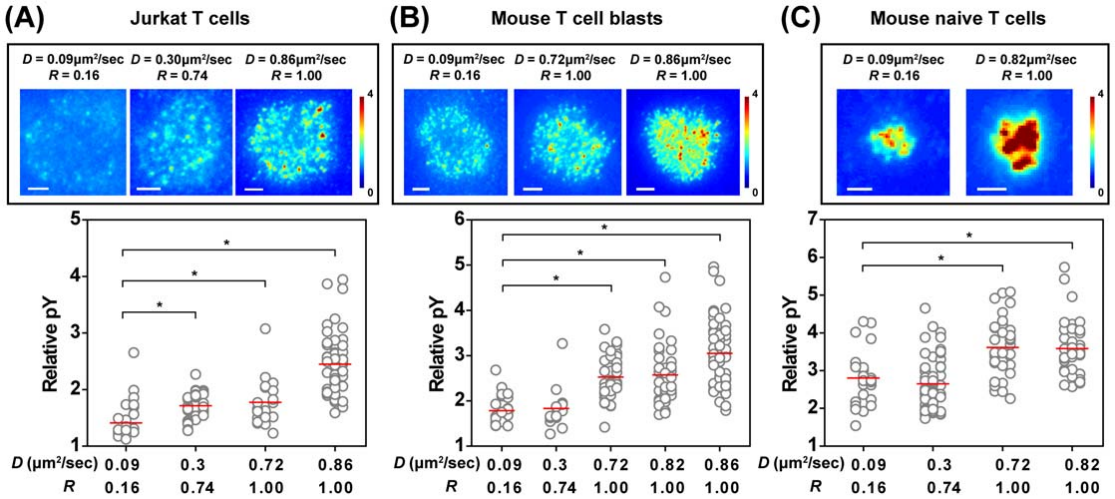
High ligand mobility promotes elevated phosphotyrosine responses. (A) Jurkat cells (B) mouse T cell blasts, and (C) mouse naïve T cells were stimulated on the indicated bilayer surfaces coated with OKT3, ICAM-1+2C11, or ICAM-1+pMHC, respectively. Cells were fixed at 5 min after cell contact, fluorescently labeled for phosphotyrosine, and imaged by TIRF microscopy. Relative phosphotyrosine intensity was determined and depicted as a heat map. Representative cells are shown (top). Scale bars = 3 µm. Relative phosphotyrosine levels in multiple cells were plotted as a function of *D* and *R* (bottom), with horizontal red lines indicating average values. Asterisks represent *p* less than 0.002 compared to low mobility membranes. N>25 cells per condition.

The relationship between ligand mobility and T cell activation is unclear. On one hand, high ligand mobility should allow T cells to accumulate more stimulatory ligand, effectively increasing antigen dose. On the other hand, there is evidence that TCR signaling is tension-based, so that low mobility conditions that restrain centripetal MC movement, might be expected to augment signaling [34]. To test the influence of ligand mobility on T cell activation, we characterized the extent of tyrosine phosphorylation and calcium flux in cells responding to bilayer surfaces with different *D* and *R*. To assess tyrosine phosphorylation, Jurkat cells were stimulated for 5 minutes on bilayer surfaces, fixed, and labeled with anti-phosphotyrosine antibody. The intensity of phosphotyrosine labeling was then quantified by fluorescence microscopy, as described in Materials and Methods. MCs containing phosphorylated proteins were observed on all stimulatory membranes, but were most prominent in cells stimulated on fluid membranes (Fig. 6A, top). Indeed, quantitation showed that cells stimulated on the fluid membrane (*D* = 0.86 µm^2^/sec, *R* = 1.00) displayed a relative phosphotyrosine intensity of 2.4, significantly higher than the values measured on the fluid-connected and fluid-confined membranes (1.8 and 1.7, respectively) (Fig. 6A, bottom). Since the level of stimulatory ligands on the different membranes was comparable (Fig. S3), we conclude that elevated phosphotyrosine levels can be attributed to increasing ligand mobility, rather than the differential protein coupling on the membranes

We further verified this finding with mouse CD4+ T cell blasts and mouse naïve T cells stimulated via 2C11 antibody and peptide-MHC Class II molecules, respectively. As in Jurkat T cells, these primary cells showed phosphorylated MCs on all stimulatory membranes (Fig. 6B–6C, top). However, cells activated on the fluid membranes exhibited stronger relative tyrosine phosphorylation than those stimulated with less mobile ligands (Fig. 6B–6C, bottom).

**Figure 7:**
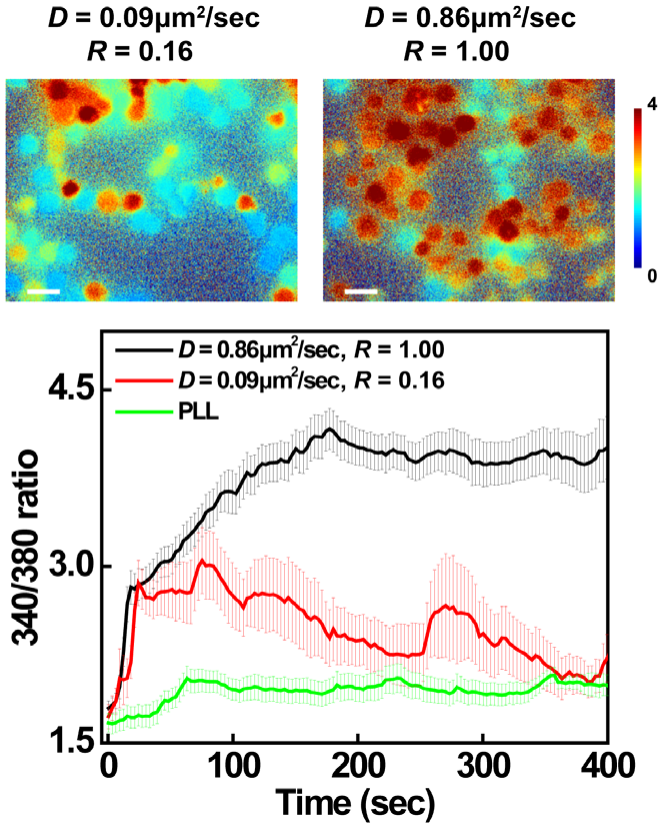
High ligand mobility triggers persistent intracellular calcium flux. Jurkat cells loaded with Fura-2 were plated on various bilayer surfaces, and intracellular calcium levels were monitored by widefield ratiometric imaging at 340/380 nm. Top, representative images of Fura-2 340/380 ratio after 20 minutes on different bilayer surfaces were depicted as a heat map (top). Scale bars = 20 µm. Bottom, average Fura-2 ratios in cells activated on bilayers with highly mobile (black) or low mobility (red) bilayers were compared to those in control cells interacting with poly-L-lysine (PLL) -coated surfaces (green). Error bars represent the standard error of the mean.

As another measure of T cell activation, intracellular calcium flux was quantified using ratiometric measurement of Fura-2 fluorescence in living cells. As Jurkat cells interacted with the fluid membranes (*D* = 0.86 µm^2^/sec, *R* = 1.00), a rapid increase in intracellular calcium was observed (Fig. 7, top). Elevated intracellular calcium levels fluctuated and persisted for several minutes, and a substantial fraction of cells still showed elevated intracellular calcium as long as 20 minutes after contact with the surface. T cells contacting the low mobility stimulatory membrane also exhibited calcium signaling initially, and this response was significantly higher than that observed in cells contacting the control poly-L-lysine coated surface (Fig. 7, bottom). In comparison with the response evoked by fluid membranes, however, the response to low mobility membranes was dramatically dampened in both magnitude and persistence. Taken together with the data on tyrosine phosphorylation, these results show that T cell activation is related to ligand mobility. The augmented cell activation on the fluid membrane could be associated with the enriched accumulation of TCR signaling complexes at the synaptic interface.

## Discussion

Supported lipid bilayers composed of a binary phospholipid mixtures have been widely used to investigate membrane heterogeneity [31], [35]. At constant temperature, surface heterogeneity and long range membrane diffusivity in the gel-fluid co-existence region vary according to the lipid composition, so that mobility within binary mixtures is governed by percolation theory [31], [36], [37]. In this study, we have used supported planar membranes composed of DPPC and DMPC. Using FRAP measurements of *D* and *R* to quantitatively describe long-range lateral movement of ligands in the presence of immobile domains, we identified lipid ratios that result in fluid, fluid-connected, fluid-confined, and low mobility membranes, and bound T cell stimulatory ligands to each of these membranes with similar efficiency and similar orientation. Using this experimental configuration, we have systematically explored the effect of varying ligand mobility on T cell activation.

Our results show that aggregation of the TCR complex within the cSMAC and centripetal movement of MCs containing key T cell signaling molecules are highly dependent on ligand mobility. Comparison of cSMAC formation in T cells interacting with fluid membranes and fluid confined membranes shows that CD3ζ centralization is delayed with fluid-connected membranes, but the final accumulation after 60 min is similar on these two surfaces. In contrast, CD3ζ accumulation plateaus at a lower level in T cells responding to fluid confined membranes. These results are consistent with a model in which the rate of TCR lateral transport is influenced by *D* while the overall level of TCR accumulated at the cSMAC depends on the connectivity of fluid regions.

The movement of MCs containing the tyrosine kinase ZAP70 toward the cSMAC also depends strongly on *D*. This likely reflects the direct association of ZAP70 with phosphorylated ITAM motifs within the TCR complex. Several factors such as cluster size [38], differential coupling to actin flow [33], and integrin engagement [34], [39] have been proposed to affect the movement of signaling MCs at the IS. Our data suggest that membrane fluidity should also be considered. In comparison with ZAP70 MCs, MCs containing the adaptor molecule SLP76 show relatively little dependence on *D*. This finding is consistent with previous work showing robust centripetal movement of SLP76 MCs in T cells responding to ligand-coated coverslips [8], [34], [40] and indicates that the mechanisms that propel or constrain these two molecules at the IS are distinct. ZAP70 MCs are likely to be associated with the plasma membrane, while there is evidence that SLP76 MCs are associated with intracellular vesicles [41], and this could explain their differential sensitivity to ligand mobility. Alternatively, these two sets of MCs may interact differently with cytoskeletal components, e.g. actin and microtubules. Indeed, recent studies have demonstrated that dynein motors essentially co-distribute with signaling complexes and TCR MCs move along microtubules within the central region of the IS [42]. In this context, it is interesting to note that retrograde flow of the actin cytoskeleton was not grossly affected by ligand mobility. Thus, ligand-dependent changes in mobility of TCR or ZAP70 cannot be attributed to slowing of the actin network.

In addition to promoting enhanced dynamics of T cell signaling proteins, we find that increased ligand mobility is associated with increased T cell activation as measured by tyrosine phosphorylation and elevated intracellular calcium. This result contrasts with previous studies, where augmented MC phosphorylation and elevated cytoplasmic calcium flux was associated with *diminished* MC movement in cells costimulated with TCR and β1 integrin ligands [34] or in cells plated on bilayer surfaces with metal grids that constrain the lateral movement of membrane components [32]. In those experimental systems, augmented signaling was proposed to result from prolonged co-distribution between TCR and active kinases at peripheral signaling sites. The mechanism by which cell activation is related to ligand mobility may be different. In T cells responding to mobile ligands, we observed an overall increase in the accumulation of CD3ζ at the IS. The large reservoir of TCR agonist could continue to generate new TCR MCs, and to recruit downstream protein tyrosine kinases, raising the pool of phosphorylated molecules and sustaining elevated intracellular calcium levels [5]. The positive correlation between ligand mobility and T cell activation cannot simply be an artifactual consequence of stimulation with anti-CD3 antibody, because similar results were obtained using peptide-loaded MHC Class II molecules and antigen-specific primary T cells. Nonetheless, the studies presented here used super-physiological concentrations of TCR agonist. In future work, it will be interesting to explore the effects of altered ligand mobility at limiting agonist dose, and to ask if the results differ depending on TCR-pMHC binding kinetics.

The reductionist system used here highlights the importance of ligand mobility and membrane heterogeneity as modulators of T cell activation. Clearly, ligand mobility in APC membranes lies somewhere between the extremes represented by the highly mobile and low mobility planar lipid bilayers. Indeed, live-cell FRAP experiments have revealed that many membrane-associated proteins exhibit relatively low long-range diffusional mobility and/or large immobile pools [43]. Moreover, cell surface molecules exhibit distinct mobility properties depending on differential cytoskeletal association, aggregation state, or lipid microdomain association. Given the strong dependence of T cell activation on ligand mobility demonstrated here, it will be important to explore the parameter of ligand mobility on the surface of APCs. Future studies should address the differences in ligand mobility among different types of APCs, and the possibility that regulated changes in APC membrane fluidity are used to modulate T cell activation in vivo.

## Materials and Methods

### Ethics Statement

All animal studies were carried out according to guidelines put forth by the NIH guide for the care and use of laboratory animals, as approved under protocol #2011-9-667 by the Children's Hospital of Philadelphia Institutional Animal Care and Use Committee.

### Plasmids, cell lines, and transfection

ZAP70-GFP cDNA was provided by Dr. L. Samelson (NIH). Human Jurkat T cells and J14 cells (SLP76-deficient Jurkat cells) stably expressing SLP76-GFP were gifts from Dr. G. Koretzky (Univ. of Pennsylvania) [39]. Jurkat T cells stably expressing actin-GFP were described previously [44]. HEK 293T cells were obtained from the American Type Cell Culture Collection (Manassas, VA). Jurkat cells were cultured in phenol red-free RPMI 1640 medium (Invitrogen, Carlsbad, CA) with 10% fetal bovine serum (Thermo Scientific HyClone, Logan, UT) and 20 mM glutamine without antibiotics in a 5% CO_2_ humidified incubator at 37°C. For transfection, cells were cultured for 24 hours at 1–2×10^5^/mL, and then resuspended at 20×10^6^/mL in serum-free RPMI medium prior to the electroporation. One single electric pulse of 310 V and 20 ms was applied to a cuvette containing 500 µL of cell solution and 20 µg of cDNA using a BTX electroporator (BTX Harvard Apparatus, St. Laurent, Quebec, Canada). Cells were used in 24–48 hours after transfection.

### Primary T cell culture and transduction

Murine CD4+ T cells were isolated from 8–12 week old C57BL/6 mice (Jackson Laboratories) or Rag1−/−:B3K506 TCR transgenic mice, which recognize the 3K peptide, an altered peptide ligand of the I-E^b^ alpha presented on I-A^b^ [45].

Lymph nodes and spleens were harvested, and single cell suspensions were subjected to negative selection with anti-MHC class II (M5/114.15.2) and anti-CD8 (53.672), followed by goat anti-rat IgG magnetic beads (Qiagen, Valencia CA). To produce T cell blasts, CD4+ T cells were stimulated for 3 days using plate-bound anti-mouse CD3 antibody (2C11) and anti-mouse CD28 antibody (PV1) at 1 µg/mL each, (both from BioXCell, West Lebanon, NH). T cells were cultured in DMEM (Invitrogen) with 10% FBS containing 100 U/ml rhIL-2 (obtained through the AIDS Research and Reference Reagent Program, Division of AIDS, National Institute of Allergy and Infectious Diseases, National Institutes of Health; human rIL-2 from M. Gately, Hoffmann-LaRoche, Nutley, NJ).

For retroviral transduction, recombinant murine ecotropic retrovirus was produced by calcium phosphate transfection of HEK 293T cells. A tissue culture dish containing 7×10^6^ cells was grown overnight and then transfected with 24 µg ZAP70-GFP cDNA and 8 µg of constructs encoding the viral envelope protein for mouse ectopic virus and the gag and pol genes. Supernatant was harvested 48 hours post-transfection and titered using NIH-3T3 cells. Each well of T cell blasts was inoculated with 1 mL of ZAP70-GFP retrovirus, 8 µg/mL hexadimethrine bromide (Sigma, St. Louis, MO) and 50 U/mL rhIL-2, and centrifuged at 2000 rpm for 1.5 hours at 24°C. T cells were harvested 48–72 hours post transduction.

### Bilayer preparation

All phospholipids and extruder accessories were purchased from Avanti Polar Lipids (Alabaster, AL). Biotinylated anti-human CD3 antibody (OKT3) was purchased from eBioscience (San Diego, CA). Biotinylated anti-mouse CD3 antibody (2C11) was purchased from BioXCell (West Lebanon, NH). Mouse ICAM-1, obtained from R&D Systems, Minneapolis, MN), was biotinlyated via Sulfo-NHS-LC-Biotin (Thermo Scientific, Rockford, IL). Supported bilayer surfaces were formed by small unilamellar vesicle (SUV) fusion on glass substrates [30]. SUVs were composed of 2 molar percent of DSPE-PEG(2000)-biotin (1,2-distearoyl-*sn*-glycero-3-phosphoethanolamine-N-[biotinyl(polyethyleneglycol)-2000]) and the desired binary mixing ratio of DMPC (1,2-dimyristoyl-*sn*-glycero-3-phosphocholine) and DPPC (1,2-dipalmitoyl-*sn*-glycero-3-phosphocholine). The mixed lipid solution was evacuated in a desiccator for at least 2 hrs. The lipid film was then rehydrated with PBS and sonicated for 30 min at 50°C. The lipid suspension was extruded through a polycarbonate filter with 50 nm pores.

Stimulatory supported bilayers were prepared essentially as described in [30]. Briefly, 300 µl of SUV solution was added to a plasma-oxidized Bioptechs delta T culture dish (Fisher, Pittsburgh, PA). After 30 min, a second injection of SUV solution was added, after which the dish was rinsed and incubated for 45 min with 0.01% BSA solution to block non-specific protein binding. Bilayers were then incubated at room temperature for 30 min with 1 µg of neutravidin (NTA, Thermo Scientific) for live-cell imaging or Texas Red-NTA (Invitrogen) for FRAP analysis. After the removal of unbound NTA, bilayer surfaces were incubated overnight with 1 µg of biotinylated OKT3 antibody for Jurkat cell studies. For studies involving primary murine T cells, NTA-bound bilayer surfaces were incubated with 0.1 µg of biotinylated mouse ICAM-1 together with 1 µg of biotinylated 2C11 antibody (for polyclonal T cell blasts) or together with 1 µg of biotinylated monomeric I-A^b^/3K complex ([45], for naive B3K506 TCR transgenic T cells). The unbound ligands were removed before the addition of cell suspension.

### FRAP measurements

Fluorescence Recovery after Photobleaching (FRAP) measurements were performed on an inverted IX71 microscope system (Olympus, Center Valley, PA). Fluorescence was collected in epi-illumination mode via a cooled EMCCD camera (Hamamatsu, Bridgewater, NJ) and recorded using HCImage software (Hamamatsu). A circular region with a radius of 23.4 µm was photobleached for 30 seconds using a mercury arc lamp. Post-bleach images were acquired and a recovery curve calculated from a standard diffusion model was fit to the data to obtain the ligand diffusion coefficient *D* and mobile fraction *R* [25].

### Atomic Force Microscopy

Atomic Force Microscopy (AFM) was performed using an Agilent 5420 microscope (Santa Clara, CA), and all accessories were purchased from Veeco (Plainview, NY). A silicon nitride probe with a spring constant of 0.01–0.06 N/m was used in contact mode, with scanning frequencies from 0.5–1.5 Hz. SUV solutions were deposited on the wafer surface and incubated at room temperature overnight. The wafer was placed in the AFM fluid cell and remained hydrated at a temperature of 37°C during the scanning process. In the fluid cell, 1 µg of NTA diluted in 300 µL PBS was added and incubated for 1 hour before AFM imaging.

### Live-cell imaging

Live-cell fluorescence imaging was performed on an inverted IX71 microscope system with a 60× 1.45NA TIRF objective (Olympus) using 50 mW 488 nm laser (Coherent, Santa Clara, CA) and appropriate neutral density filters (Thorlabs, Newton, NJ). Wild-type Jurkat T cells transiently transfected with ZAP70-GFP or J14 cells stably expressing SLP76-GFP were maintained at 37°C using Bioptechs objective and culture dish heaters (Fisher). Images were collected every 0.1 second using a cooled EMCCD camera (HAMAMATSU, Bridgewater, NJ), and MC movement was analyzed with MATLAB (MathWorks, Natick, MA), as previously described [39]. MC track velocity was defined as the total distance traveled by a given MC, divided by its total track duration. MC net displacement was computed as the shortest distance between the position where a single MC first appeared and the position where that MC disappeared from tracking.

### Immunostaining

For immunofluorescence microscopy of fixed cells, Jurkat T cells were first resuspended at 2–5×10^6^/mL in phenol red-free RPMI medium without serum, and allowed to interact with stimulatory surfaces for the indicated times. Cell were fixed with 4% formaldehyde (Fisher), permeabilized with 0.5% of Triton X-100 (Roche, Basel, Switzerland) and blocked with 10% BSA (Sigma) in PBS. For phosphotyrosine labeling, cells were incubated with anti-pY clone 4G10 primary antibody (Millipore, Billerica, MA) followed by goat anti-mouse Alexa568 secondary antibody (Invitrogen). For cSMAC labeling, cells were incubated with FITC-conjugated anti-CD3ζ antibody (Antibodies-online). Immunofluorescence intensity was quantified via ImageJ. The relative phosphotyrosine and CD3ζ levels were defined as the ratio of the fluorescence intensity over the entire cell-surface interface to the intensity of background (regions devoid of cells).

### Calcium imaging

Calcium imaging was done essentially as previously described [46]. Briefly, Jurkat T cells were resuspended at 2×10^6^/mL and incubated with 0.5 µM Fura-2 (Invitrogen) in imaging buffer (155 mM NaCl, 4.5 mM KCl, 2 mM CaCl_2_, 1 mM MgCl_2_, 10 mM glucose, 10 mM HEPES, pH 7.4) at room temperature for 20 min. Cell suspensions were then washed with imaging buffer, added to warmed stimulatory surfaces and imaged every 3 s by sequential illumination with 340 and 380 nm lasers. Acquisition was done on a Leica DMI6000 microscope using an Orca-03G camera (Hamamatsu) and a 40× oil objective, and images were analyzed with Metafluor (Molecular Devices, Sunnyvale, CA).

## Supporting Information

Figure S1 FRAP responses of bilayers composed of binary lipid mixtures. Long-range diffusion coefficient and recovery fraction of Texas-red NTA were determined by fluorescence recovery after photobleaching (FRAP) measurements. (A) One representative FRAP experiment performed on DMPC bilayers. Scale bar = 5 µm. (B) Representative fluorescence recovery data points obtained from supported lipid bilayer surfaces containing 3/7 DMPC/DPPC (black squares), 5/5 DMPC/DPPC (red circles), 7/3 DMPC/DPPC (green upright triangles), 8/2 DMPC/DPPC (blue inverted triangles) or DMPC (cyan diamonds) with constant 2% DSPE-PEG2000-biotin in the presence of neutravidin-Texas Red at 37°C. Best fit curves for each condition are shown in gray lines.
(TIF)

Figure S2 Addition of NTA introduces inhomogeneity in bilayers containing 70% DMPC. Representative 3 µm×3 µm AFM images of 70% DMPC and 30% DPPC supported membranes incubated without (A) or with (B) NTA at 37°C. (C) Surface height along the yellow dashed lines in A (green) and B (black).
(TIF)

Figure S3 The amount of protein coupled on membranes was not influenced by ligand mobility. Supported bilayer surface decorated with 2% mouse anti-human CD3 antibody was incubated with anti-mouse Alexa568 antibody to quantify the distribution of coupled protein. Immunofluorescence intensity from the region of interest was normalized to the maximum intensity among all the surfaces in each trial. Average normalized intensity for each surface was analyzed from at least 9 different regions and three independent trials. Error bars represent the standard error of the mean. N>20 areas per condition.
(TIF)

Figure S4 ICAM1 ligation alone does not stimulate mouse T cell blasts. (A) CD4+ mouse T cell blasts were added to bilayers coated with ICAM1 only (top) or ICAM1 together with 2C11 (bottom). Cells were then fixed, labeled with anti-phosphotyrosine, and imaged by TIRF microscopy. (B) CD4+ mouse T cell blasts were transduced with ZAP70-GFP, stimulated as in A, and imaged by TIRF microscopy. Neither effective tyrosine phosphorylation nor ZAP70 MC formation was observed on supported bilayers containing ICAM-1 only. Scale bars = 5 µm.
(TIF)

Video S1 Live-cell tracking of ZAP70-GFP in Jurkat T cells stimulated by OKT3 on low mobility membranes. Video length = 90 seconds.
(AVI)

Video S2 Live-cell tracking of ZAP70-GFP in Jurkat T cells stimulated by OKT3 on fluid membranes. Video length = 90 seconds.
(AVI)

Video S3 Live-cell tracking of SLP76-GFP in Jurkat T cells stimulated by OKT3 on low mobility membranes. Video length = 90 seconds.
(AVI)

Video S4 Live-cell tracking of SLP76-GFP in Jurkat T cells stimulated by OKT3 on fluid membranes. Video length = 90 seconds.
(AVI)

Video S5 Live-cell tracking of ZAP70-GFP in Mouse CD4+ T cell blasts stimulated by 2C11 on low mobility membranes. Video length = 300 seconds.
(AVI)

Video S6 Live-cell tracking of ZAP70-GFP in Mouse CD4+ T cell blasts stimulated by 2C11 on fluid membranes. Video length = 160 seconds.
(AVI)
