# Supplementary figures and images for: Ligand Mobility Modulates Immunological Synapse Formation and T Cell Activation
Source: PLoS One. 2012 Feb 22;7(2):e32398. doi: 10.1371/journal.pone.0032398 (PMC3284572; doi:10.1371/journal.pone.0032398)

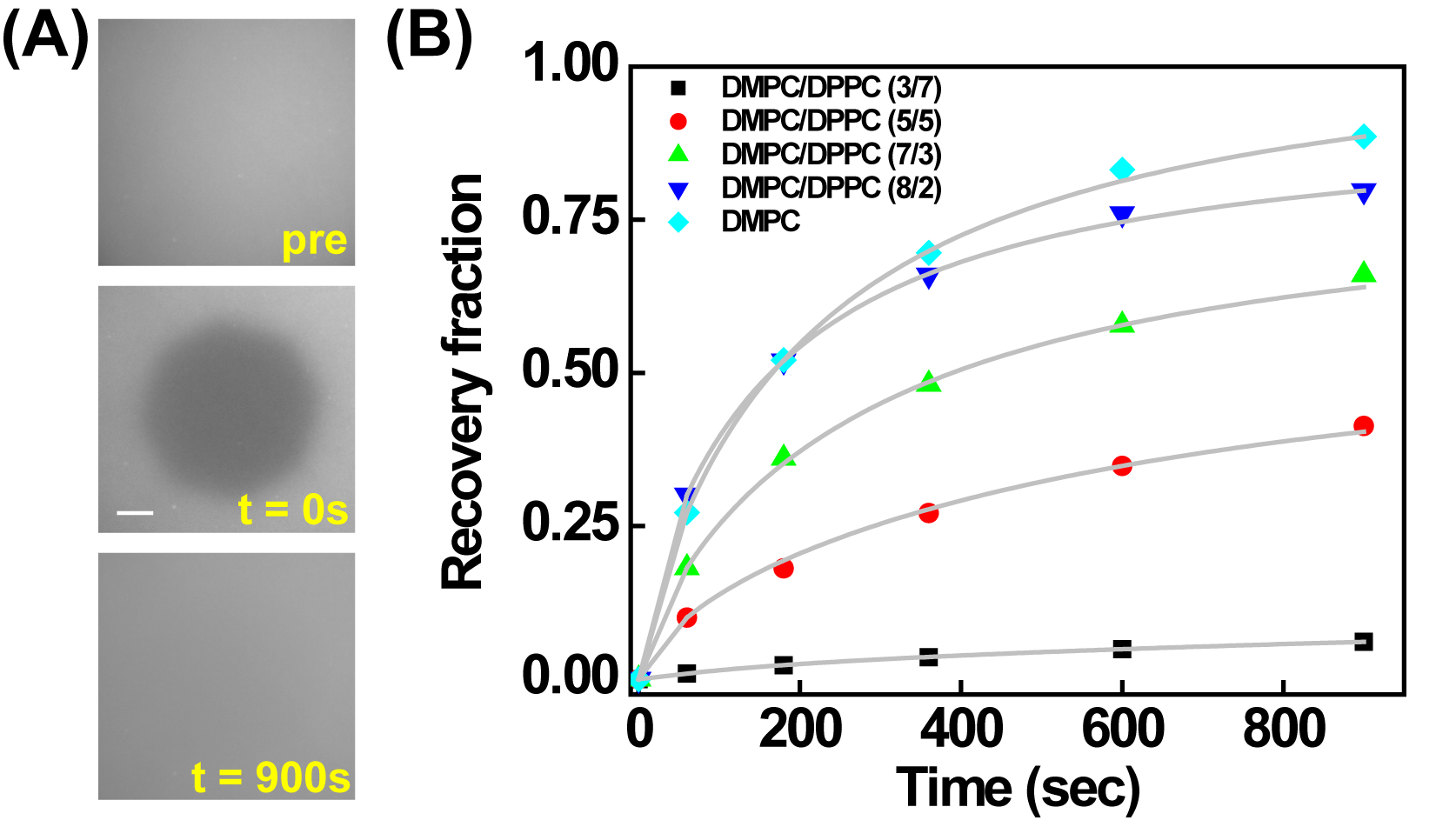

Supplement: Figure S1 — FRAP responses of bilayers composed of binary lipid mixtures. Long-range diffusion coefficient and recovery fraction of Texas-red NTA were determined by fluorescence recovery after photobleaching (FRAP) measurements. (A) One representative FRAP experiment performed on DMPC bilayers. Scale bar = 5 µm. (B) Representative fluorescence recovery data points obtained from supported lipid bilayer surfaces containing 3/7 DMPC/DPPC (black squares), 5/5 DMPC/DPPC (red circles), 7/3 DMPC/DPPC (green upright triangles), 8/2 DMPC/DPPC (blue inverted triangles) or DMPC (cyan diamonds) with constant 2% DSPE-PEG2000-biotin in the presence of neutravidin-Texas Red at 37°C. Best fit curves for each condition are shown in gray lines. (TIF) [file pone.0032398.s001.tif]

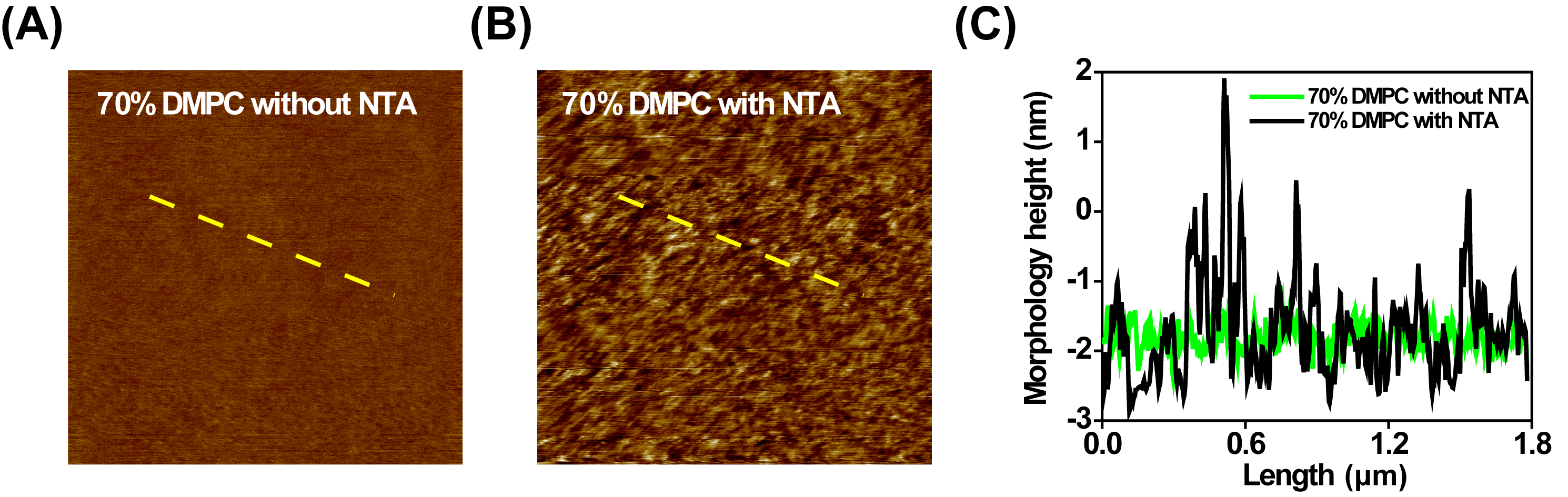

Supplement: Figure S2 — Addition of NTA introduces inhomogeneity in bilayers containing 70% DMPC. Representative 3 µm×3 µm AFM images of 70% DMPC and 30% DPPC supported membranes incubated without (A) or with (B) NTA at 37°C. (C) Surface height along the yellow dashed lines in A (green) and B (black). (TIF) [file pone.0032398.s002.tif]

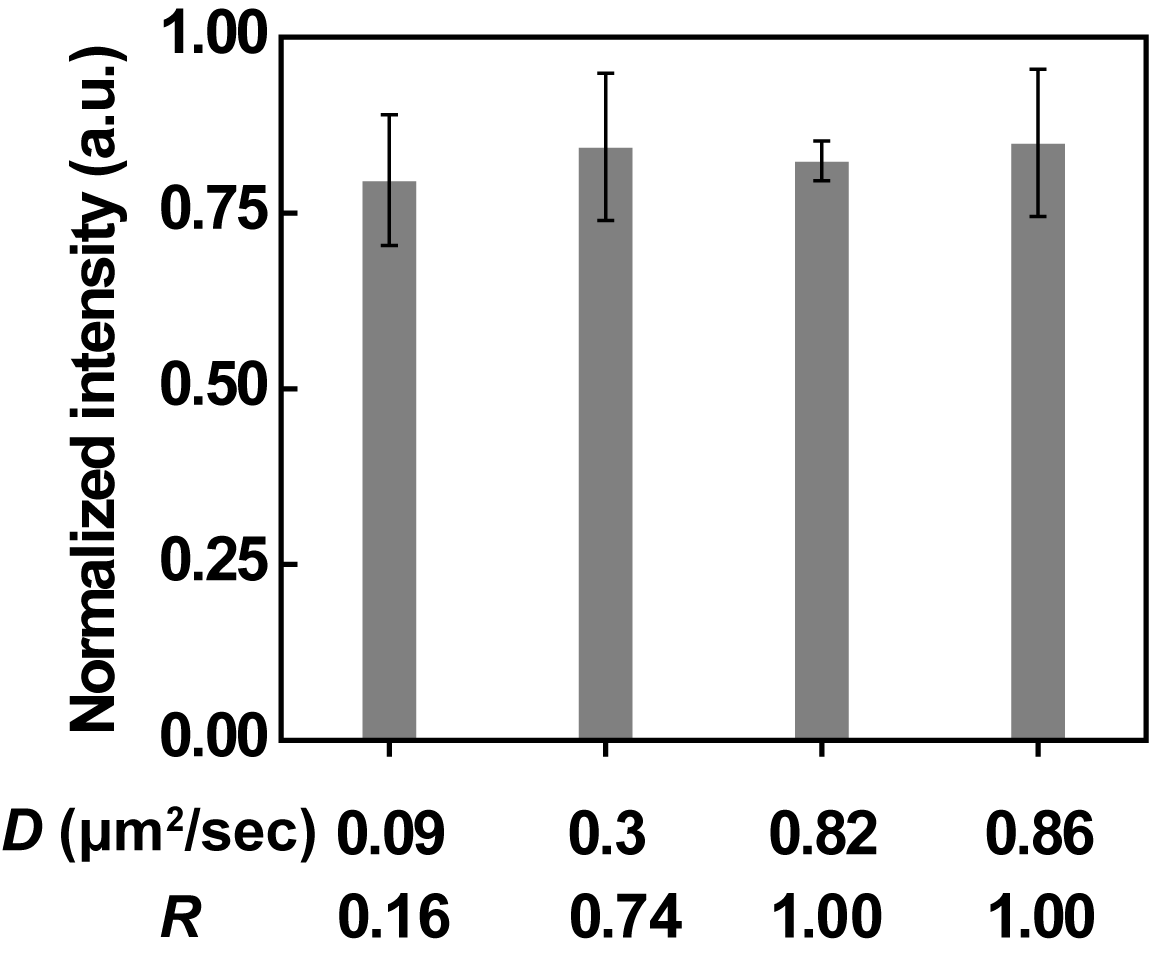

Supplement: Figure S3 — The amount of protein coupled on membranes was not influenced by ligand mobility. Supported bilayer surface decorated with 2% mouse anti-human CD3 antibody was incubated with anti-mouse Alexa568 antibody to quantify the distribution of coupled protein. Immunofluorescence intensity from the region of interest was normalized to the maximum intensity among all the surfaces in each trial. Average normalized intensity for each surface was analyzed from at least 9 different regions and three independent trials. Error bars represent the standard error of the mean. N>20 areas per condition. (TIF) [file pone.0032398.s003.tif]

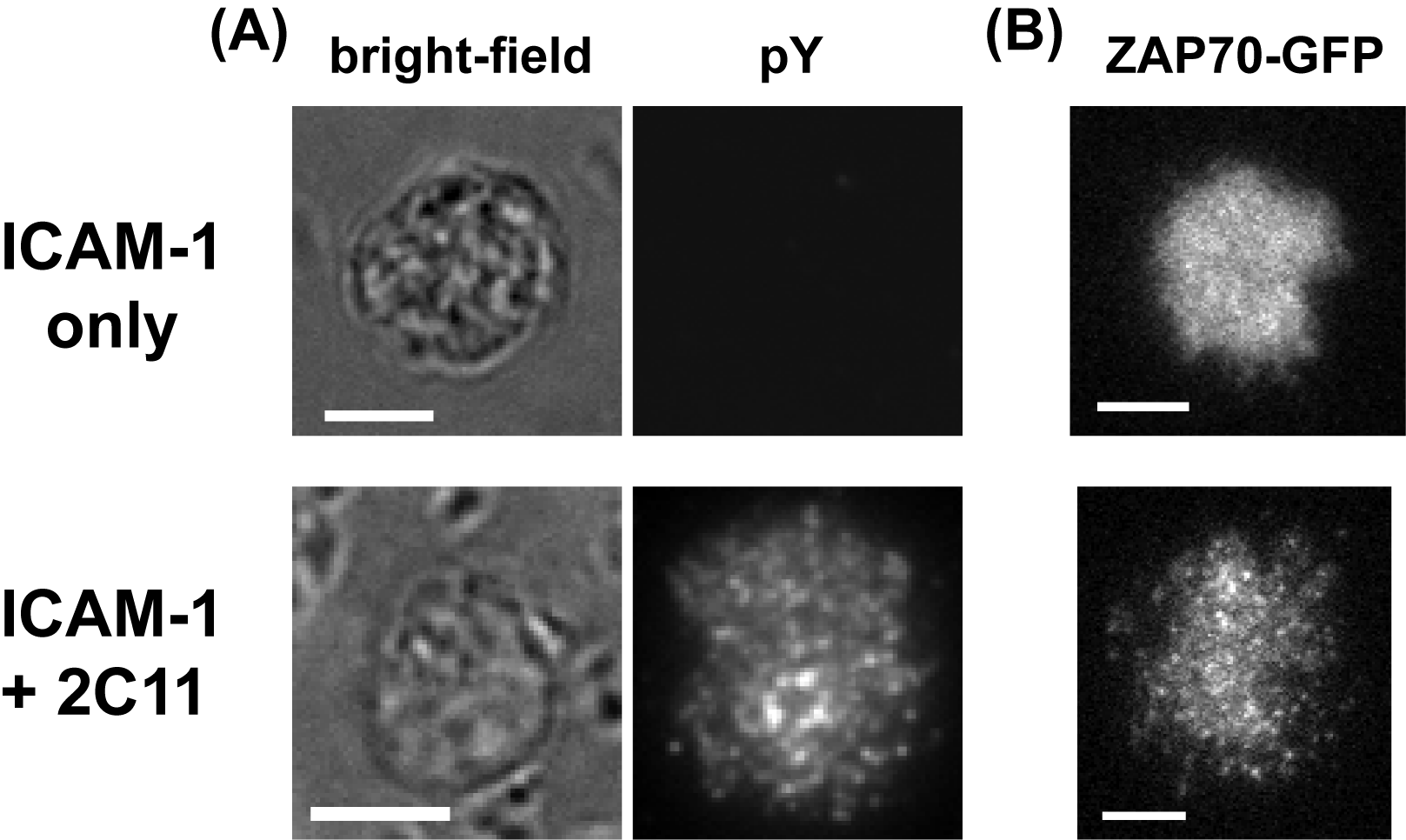

Supplement: Figure S4 — ICAM1 ligation alone does not stimulate mouse T cell blasts. (A) CD4+ mouse T cell blasts were added to bilayers coated with ICAM1 only (top) or ICAM1 together with 2C11 (bottom). Cells were then fixed, labeled with anti-phosphotyrosine, and imaged by TIRF microscopy. (B) CD4+ mouse T cell blasts were transduced with ZAP70-GFP, stimulated as in A, and imaged by TIRF microscopy. Neither effective tyrosine phosphorylation nor ZAP70 MC formation was observed on supported bilayers containing ICAM-1 only. Scale bars = 5 µm. (TIF) [file pone.0032398.s004.tif]
